# A novel way to detect correlations on multi-time scales, with temporal evolution and for multi-variables

**DOI:** 10.1038/srep27707

**Published:** 2016-06-13

**Authors:** Naiming Yuan, Elena Xoplaki, Congwen Zhu, Juerg Luterbacher

**Affiliations:** 1 Department of Geography, Climatology, Climate Dynamics and Climate Change, Justus Liebig University Giessen, 35390 Giessen, Germany; 2 Chinese Academy of Meteorological Science, Beijing, 100081, China; 3 Centre for International Development and Environmental Research, Justus Liebig University Giessen, 35390 Giessen, Germany

## Abstract

In this paper, two new methods, Temporal evolution of Detrended Cross-Correlation Analysis (TDCCA) and Temporal evolution of Detrended Partial-Cross-Correlation Analysis (TDPCCA), are proposed by generalizing DCCA and DPCCA. Applying TDCCA/TDPCCA, it is possible to study correlations on multi-time scales and over different periods. To illustrate their properties, we used two climatological examples: i) Global Sea Level (GSL) versus North Atlantic Oscillation (NAO); and ii) Summer Rainfall over Yangtze River (SRYR) versus previous winter Pacific Decadal Oscillation (PDO). We find significant correlations between GSL and NAO on time scales of 60 to 140 years, but the correlations are non-significant between 1865–1875. As for SRYR and PDO, significant correlations are found on time scales of 30 to 35 years, but the correlations are more pronounced during the recent 30 years. By combining TDCCA/TDPCCA and DCCA/DPCCA, we proposed a new correlation-detection system, which compared to traditional methods, can objectively show how two time series are related (on which time scale, during which time period). These are important not only for diagnosis of complex system, but also for better designs of prediction models. Therefore, the new methods offer new opportunities for applications in natural sciences, such as ecology, economy, sociology and other research fields.

Detecting cross-correlations between two signals is the most usual way to diagnose and understand a complex system. The simplest method is the traditional Pearson Cross-Correlation Analysis, which has been widely used in both natural systems such as climate system, ecosystem, etc.^1^,^2^,^3^,^4^, and social systems such as economy or finance^5^,^6^. In statistics, Pearson Correlation Coefficient (PCC) is one of the most popular statistical measure, and is frequently discussed in almost all fields. For example, in climatology, PCC is widely used for dynamical diagnosing and climate forecasting^7^,^8^,^9^. In economics, PCC is applied to establish economical models^10^,^11^. However, signals obtained in nature are usually affected by many nonlinear processes, and sometimes external forcings^12^,^13^,^14^,^15^. Duo to the nonlinearity and nonstationarity, traditional PCC is not in all cases appropriate and it can provide erroneous results. Therefore, to better describe the relations between two signals, besides the simple PCC value, more detailed information are needed.

In this study, we aim to propose a new way to describe correlations. In order to exclude the effects of nonlinear process and external forcing (such as anthropogenic influence on global warming), the way we detect correlations should be able to i) provide cross-correlations on different time scales; ii) extract “intrinsic” relationships between two considered time series with possible influences of other common-coupled signals removed; and iii) show the time evolution of cross-correlations. For the first point i), as discussed in previous studies^16^,^17^,^18^,^19^, Detrended Cross-Correlation Analysis (DCCA) may be a solution. By calculating the DCCA coefficient *ρ*_*DCCA*_, one receives the cross-correlation levels between two variables on different time scales. This is important, especially for variables with typical periods. For instance, when the relations between the Summer Rainfall over the Yangtze River (SRYR, see Fig. 1a) and the previous winter sea surface temperature anomalies over the Nino3 region (Nino3-SSTA, see Fig. 1b) are studied, non-significant correlations are found between them over the period 1951–2012 (PCC = 0.19, see Fig. 1b). However, low PCC does not necessarily mean there are no relations, as it is well known that the SRYR is teleconnected with the previous winter central-eastern sea surface temperature. The reason why non-significant correlations between SRYR and Nino3-SSTA are found may be due to the interference of relationships among different time scales. In other words, when studying the potential effects of Nino3-SSTA on the SRYR, we may need to focus on the typical time scales of Nino3-SSTA, where the potential influences from other time scales may be minimized. In^20^, by using DCCA, the authors studied the relations between SRYR and winter Nino3-SSTA. Significant correlations between SRYR and Nino3-SSTA are found on the time scale of about 4–7 years, thus, the winter central-eastern SSTA has the main contribution to the variation of SRYR. Therefore, DCCA provides more detailed and reasonable information than the PCC-value. Although this consideration is limited to the analysis of only *two* time series, this method still can help to address an improved way for correlation detecting, that is, to discuss correlations on multi-time scales.

There are traditional methods such as filter methods (low-pass, high-pass, and band-pass, etc.)^21^,^22^, the Cross-Spectrum Analysis (CSA)^4^,^23^, as well as the Cross Wavelet Transformation method (CWT)^24^, that are used to study correlations on multi-time scales. However, for the filter methods it is necessary to determine the time scales of interest first from spectrum analysis, then low-pass, high-pass, or band-pass filtering can be performed. For CSA, it requires the data to be stationary with no external trends, which are rare in nature. As for the CWT, it works similar as DCCA, but limited to the analysis of only two time series. Regarding the second point ii) mentioned in the last paragraph, when two time series are influenced by common external influences, the real relationships between them may not be revealed by CWT. Although DCCA is not able to remove the potential influences of common-coupled external factors either, as discussed in^20^, it can be easily extended by combining partial-correlation technique, as Detrended Partial-Cross-Correlation Analysis (DPCCA). Because of the partial-correlation technique, DPCCA is applicable in multi-coupled time series, and the so-called “intrinsic” relations between two considered time series can be calculated with potential influences of other coupled signals removed. For example, no significant relations can be found between SRYR and PDO on the PDO-typical time scales when DCCA is applied. However, after removing the effects of Nino3-SSTA (El Ni*ñ*o), significant relation between SRYR and PDO is found^20^. Therefore, DPCCA has advantages in dealing with multi-coupled time series.

To study cross-correlations on multi-time scales, one can apply DCCA (*ρ*_*DCCA*_) for two time series, and DPCCA (*ρ*_*DPCCA*_) for multi-coupled signals. However, it is important to note that both DCCA and DPCCA are not able to provide information on temporal evolution, which is the third point iii) we wish to address here. Temporal evolution of cross-correlation is important because normally complex systems are characterized by non-stationarity^25^,^26^,^27^. Instantaneous correlations over specific time intervals can be different from the correlations obtained over the whole time span. Recent work from climate science shows that, the correlations between southern China summer rainfall and equatorial center Pacific SST anomalies can be opposite in two time periods (1951–1971 and 1978–1998)^28^, which as a result, the correlations calculated over the whole time span may cannot provide useful information. To illustrate this issue more clearly, we applied DCCA to two pairs of artificially generated time series: {*x*_1_, *x*_2_} and {*x*_1_, *x*_3_}. The three time series are designed as following,


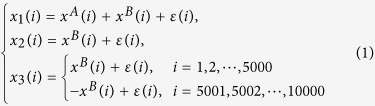


where *x*^*A*^(*i*), *x*^*B*^(*i*) are two periodical signals with different periods (*x*^*A*^(*i*) : 1000; *x*^*B*^(*i*) : 100), while *ε*(*i*) represents white noise. All the signals have length of 10000. The three time series we use for DCCA are generated according to the different combination shown above. The DCCA coefficients *ρ*_*DCCA*_ for pair {*x*_1_, *x*_2_} and pair {*x*_1_, *x*_3_} are shown in Fig. 2a,c, respectively. In Fig. 2a, since both {*x*_1_} and {*x*_2_} have the component of {*x*^*B*^}, high correlation level on time scale of 100 are found, which confirms the ability of DCCA in detecting correlation on multi-time scales. However, in Fig. 2c, the high correlation on time scale of 100 disappeared. This is because {*x*_3_} has only the component of {*x*^*B*^} for *i* = 1 to 5000. For the second half, {*x*_3_} is generated by combining negative {*x*^*B*^}. When calculating correlations over the whole length, the correlation signal will be offset. In this case, temporal evolution of cross-correlation is needed. Figure 2b,d show the detailed correlations (on time scale of 100) over different time intervals. For the pair {*x*_1_, *x*_3_}, there are high positive correlations over the first half (*i* = 1 to 5000), while high negative correlations over the second half (*i* = 5001 to 10000). Therefore, low correlations over the whole time span (Fig. 2c) do not necessarily mean there is no relations between the considered time series (Fig. 2d).

In this study, we aim to further generalize DCCA and DPCCA to time dimension, as Temporal evolution of DCCA (TDCCA) and Temporal evolution of DPCCA (TDPCCA), and finally establish a new system for correlation analysis. Two climatological examples are used to illustrate the ability of the new methods in providing temporal evolution of cross-correlation. One example is for the analysis of only two time series, we apply DCCA with information of temporal evolution included (TDCCA). The other one is for the analysis of multi-coupled time series, where the ability of TDPCCA are shown. With these methods, correlations on different time scales and over different time periods can be calculated, which are useful information for further studies of complex systems. In the end of this paper, detailed introduction of the new methods is made (see the “Method” section).

## Results

### Temporal evolution of DCCA (TDCCA)

**Figure 3:**
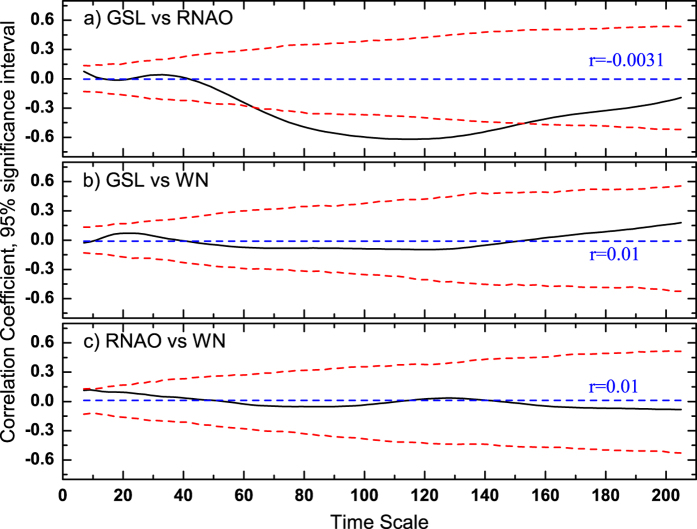
DCCA cross-correlation coefficients between Global Sea Level (GSL) and Reconstructed North Atlantic Oscillation (RNAO). The black curves are the DCCA results, the red dashed line are the threshold of 95% confidence intervals calculated from Monte-Carlo tests, while the blue dashed line represents the Pearson’s correlation coefficient. (**a**) shows the results for GSL and winter RNAO covering the period 1700–2001. For comparison, (**b**) shows the results for GSL and White Noise (WN), and (**c**) the results for RNAO and WN.

To illustrate the ability of TDCCA, we will discuss the relationships between the annual Global Sea Level record (GSL, 1700–2001)^29^ and a Reconstruction of the winter North Atlantic Oscillation (RNAO, 1700–2001)^30^,^31^. Both records have been studied in^32^ by Multi-Scale Dynamical Analysis (MSDA). In MSDA, one can calculate the oscillation patterns of different records through their accelerations on different time scales over different time periods. In^32^, it is found that the GSL and RNAO have negatively correlated oscillation patterns on time scales of 30~110 years, which indicates the global sea level may decrease when winter NAO is positive. However, the Pearson cross-correlation coefficient over the full period is around 0 (PCC = −0.003). Therefore, it is necessary to check how the two time series are connected on different time scales. Calculating the DCCA cross-correlation coefficients *ρ*_*DCCA*_ between GSL and RNAO over the 1700 to 2001 period shows significantly negative cross-correlations on time scales between 60 and 140 years (Fig. 3a). To confirm this result, we also applied the same analysis to GSL and white noise (Fig. 3b), as well as to RNAO and white noise (Fig. 3c). The Pearson Correlation Coefficients are around zero for both cases, and there are no typical time scales where significant correlations can be found. Therefore, we have confidence that there are indeed connections between GSL and RNAO on time scales of 60 to 140 years. This finding is in agreement with the previous study^32^. However, compared with MSDA which calculates “accelerations” for each record seperately, DCCA is able to calculate the cross-correlation levels of two records directly on different time scales (see equation (7) in the “Method” section, also refer to the explanations in^19^), therefore, it is more straightforward for the detection of correlations.

**Figure 4:**
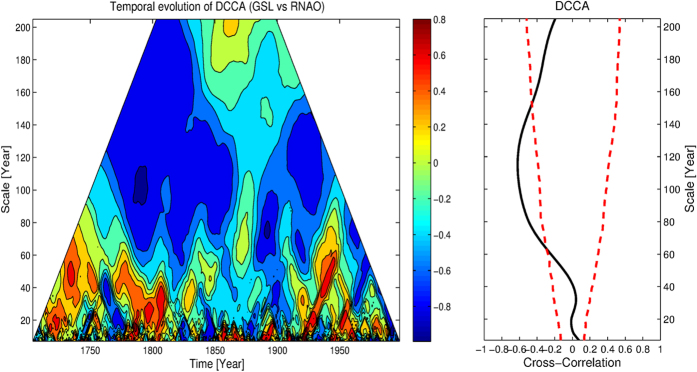
Temporal evolution of correlations between GSL and RNAO, on different time scales. Left hand side (LHS) is the TDCCA result for GSL and RNAO. Right hand side (RHS) is the DCCA results (the same as Fig. 3a). On time scales of 60 to 140 years, significant correlations are found. But over time, a “halfway” break off is visible between the years from 1865 to 1875, indicating unstable negative correlations over time.

**Figure 5:**
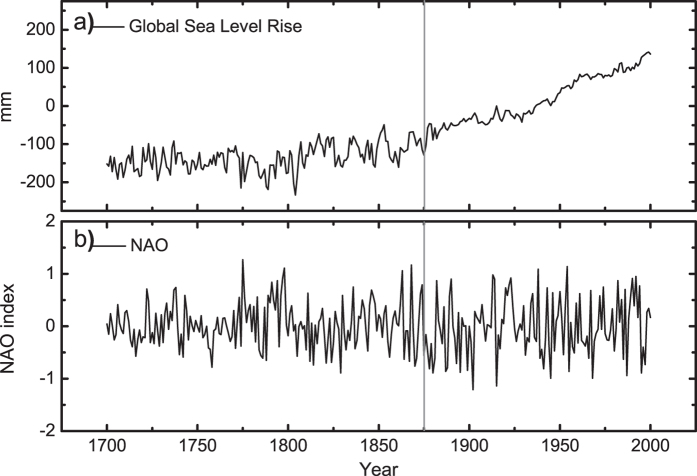
Global Sea Level (GSL) records and the Reconstructed winter North Atlantic Oscillation (RNAO) index. (**a**) GSL, (**b**) RNAO. The grey line indicates the year of 1875.

However, we would like to stress that the results shown in Fig. 3 are basically “averaged” correlations over the whole time span (1700–2001). Considering the non-stationarity of the climate system, instantaneous correlations over specific time intervals may vary over time. In this study, we generalized DCCA to time dimension, as TDCCA, and the results are shown in Fig. 4a. As expected, on time scale of 60–140 years, there is a negative-correlation band. However, the correlations are not stationary over time. A dividing zone around the year from 1865 to 1875 can be found, when very low (or no) correlations are found. The true reason for the “halfway” break off of the negative-coorelation band is not clear. It may be due to many reasons such as the changes of data quality, or a change of variability in the GSL records before and after the grey dividing line (1875), see Fig. 5a. Meanwhile, we may also attribute the “halfway” break off to the modulatory effects of some unknown background factors, which may have large cycles and the “halfway” break off we found in Fig. 4 may simply be a part of the cycle. From this example, we emphasize the importance of determining the temporal evolution when studying cross-correlations, and further illustrate the ability of TDCCA here. With the information provided by TDCCA, a more detailed diagnosis on the system will be achieved, which is important to better understand the whole system.

### Temporal evolution of DPCCA (TDPCCA)

**Figure 6:**
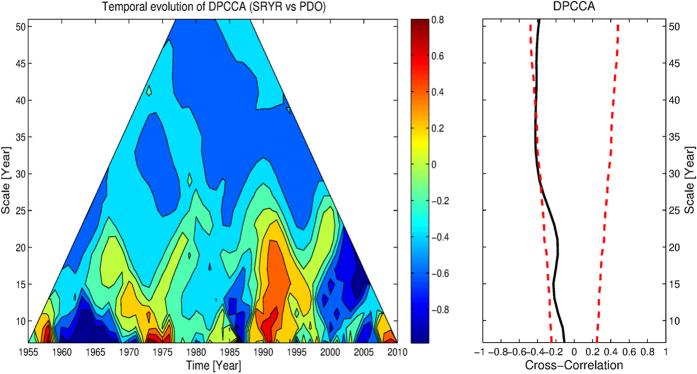
Temporal evolution of the correlations between SRYR and winter PDO, on different time scales. On the left hand side (LHS) is the TDPCCA result for SRYR and PDO, where the possible effects of Nino3-SSTA have been removed. Same as Fig. 5, on the right hand side (RHS) is the DPCCA results. One can see that after the end of 1970s, there are more significant correlations between the summer rainfall over the middle-lower reaches of Yangtze River and previous winter-time PDO index.

To address the potential reasons for the “halfway” break off of the negative correlations between GSL and winter NAO, we mentioned that it may be due to a third unknown factor which plays a role as the background and modulates the correlation pattern. To address this issue, we can combine DCCA with partial cross-correlation analysis, proposed as DPCCA^20^. Here in this study, we further generalize DPCCA to time dimension, as TDPCCA, and provide not only “intrinsic” correlations of different time scales, but also correlation evolutions over time. To illustrate the ability of TDPCCA, we revisit the relationship between the Summer Rainfall over Yangtze River (SRYR) and the previous winter-time Pacific Decadal Oscillation index (PDO) covering the period 1951–2012. As discussed in^20^, after the influence of Nino3-SSTA removed, the DCCA coefficient *ρ*_*DCCA*_ will be modified into DPCCA coefficient *ρ*_*DPCCA*_ (see Fig. 6, right hand side, RHS), and significant correlations can be found on time scales of 30–35 years. However, the relationships between SRYR and PDO may be not stationary. Thus, the “intrinsic” correlations obtained from DPCCA may not be able to reflect the true relations between them over the full time period. In fact, there are many studies showing that the climate regime has been changed around the end of 1970s^33^,^34^. Also the summer rainfall over the Yangtze River was relatively low before 1979 followed by higher precipitation sums afterwards^35^. This regime shift appeared accompanying the global scale interdecadal shift, therefore it has been attributed to several possible sources, including the weakening of east Asian summer monsoon^34^,^36^, the enlarged, intensified and extended southwestward Subtropical Northwestern Pacific High (SNPH)^35^, the changes of sea surface temperatures (SSTs) in the Indian Ocean and Atlantic Ocean^37^,^38^,^39^, and even the changes of snow cover over Tibetan Plateau^40^. Among all the possible sources, the Pacific Decadal Oscillation (PDO)^41^,^42^, is considered as a major contributor of the regime shift in the 1970s. From 1976–1977, PDO changed to a “warm” phase. The concurrent changes in sea surface temperature, sea level pressure, and land precipitation, etc., may thus be related to the changes of PDO. By using TDPCCA we revealed how the cross-correlations between SRYR and PDO vary over time. Although on time scales of 30–35 years there are correlations with the same sign, a clear division still can be found at the end of 1970s (Fig. 6, left panel). Before the late 1970s, there are negative cross-correlations between the two considered time series, but not as strong as after 1980. Thus, there are significant correlations between SRYR and PDO on time scale of 30–35 years, and the correlations are higher during the past 30 years. The change in correlations between SRYR and PDO might be related to the regime shift discussed above, which shows the complexity of the climate system, and further emphasized the necessity of detecting correlations on multi-time scales, with temporal evolution, and for multi-variables. With all these information, better diagnose of the mechanisms and even prediction models may become possible. For instance, when establishing a model to predict the SRYR, the previous winter-time PDO index can act as an predictor. From our results, it is better to only use PDO index from the past 30 years, when the correlations are more significant.

## Discussion

The use of TDCCA and TDPCCA can help to detect correlations i) among multi-time series, ii) on different time scales, and iii) further over different time period. However, open issues include the linear assumptions of partial cross-correlation analysis^20^, as well as testing for significance, should also be paid special attention. In this section, we will discuss how to test the significance and finally summarize the steps of how to use the new methods to detect correlations.

**Figure 7:**
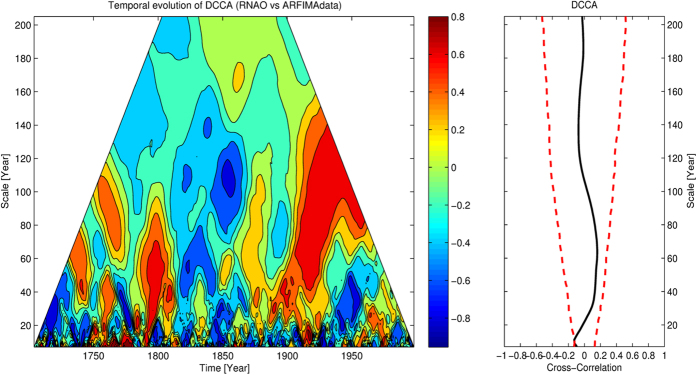
Temporal evolution of the correlations between winter RNAO and ARFIMA data, on different time scales. On the left hand side (LHS) is the TDCCA result for RNAO and ARFIMA data, while on the right hand side (RHS) is the DCCA results. As one can see, although there should be no correlations between RNAO and ARFIMA data, there are still some moments (especially on small time scales) when very big correlations by chance can be found in the TDCCA.

In DCCA and DPCCA, we determine the threshold of 95% significance by applying Monte-Carlo test. As shown in Figs 5 and 6 (red dashed lines, RHS), by shuffling the data and repeating DCCA/DPCCA for 10,000 times, the threshold of 95% significance on each time scale can be calculated. As for TDCCA and TDPCCA, it is challenging, especially when estimating significance on small time scales. For instance, we apply TDCCA to study the relationships between RNAO and randomly forced ARFIMA data (ARFIMA, autoregressive fractionally integrated moving average, which is used to model the temporal persistence of GSL^43^). Since the ARFIMA data is generated from random series (see “Method” section), unlike the TDCCA result between RNAO and GSL (see Fig. 4), there should be no correlations between RNAO and the ARFIMA data. However, as shown in Fig. 7, on small time scales (e.g., time scale smaller than 20 years), positive (negative) cross-correlations can be very high (low) during specific times. We believe the high (low) positive (negative) correlations are originated by chance, as the shorter time series we analyze, the higher uncertainties the calculated correlations have. In our methods, since we aim to study correlations among multi-time scales, the time series of interest will inevitably be divided into sliding windows of different size (time scale *s*, see the “Method” section). As a result, there is a risk of increased uncertainty in time window of small size. When applying TDCCA/TDPCCA, one may easily ask whether the cross-correlations found on small time scales indeed come from dynamical relations, or only from statistical uncertainties. To answer this question, we emphasize that although there may be “strong correlations” calculated from random noises, their signs normally alter from positive (negative) to negative (positive) frequently and abruptly, as shown in Fig. 7. However, in the cases with physical meanings, the real correlations should be able to maintain the sign unchanged for a period at least longer than the corresponding time scale *s*, as shown in Figs 4 and 6. Therefore, to perform significance test in TDCCA and TDPCCA, we need to implement another criterion. That is, the period when the correlation coefficients have the same sign must be longer than the corresponding time scale *s*. Below, we summarize the necessary steps to apply the new methods for detecting correlations:

1. Apply TDCCA/TDPCCA to the two time series of interest, to check the temporal evolution of correlation on different time scales. For a given time scale *s*, if there is a period when the sign of correlations is unchanged longer than *s*, a further detailed DCCA/DPCCA analysis specially on this time period is suggested.
2. Apply DCCA/DPCCA to the time period as suggested in i). If Monte-Carlo testing returns significant cross-correlation, the two time series are considered as significantly correlated on this typical time scale and over this specific time period.
3. Finally we determine on which time scales and during which time period the two time series are significantly correlated.

## Conclusion

In this work, we generalized DCCA and DPCCA to time dimension, and proposed two new methods, TDCCA and TDPCCA, for correlation detecting. When the correlation of only two time series needs to be studied, we can apply TDCCA (see the first example). While when there are multi-coupled time series (more than two time series) being studied, TDPCCA is needed (see the second example). With these two methods, we can not only study the correlations of two time series on multi-time scales, but also check the changes of the correlations over time. Therefore, the new methods are useful and can provide more detailed correlation information.

Regarding the significance of the results from TDCCA and TDPCCA, we find besides using Monte-Carlo simulation, it is necessary to check whether the period when the correlation coefficients have the same sign is longer than the corresponding time scale *s*, or not. Following this criterion, we finally combined DCCA/DPCCA, TDCCA/TDPCCA together as a new correlation detection system, and summarized the steps of how to use the new methods to detect correlation.

The obvious advancements and improvements of the proposed methods compared to other methods such as the filter methods, the Cross-Spectrum Analysis (CSA), and the Cross Wavelet Transformation method (CWT) is, that they are able to i) study the cross-correlations of two time series on different time scales objectively; ii) identify the temporal evolution (non-staionarity) of the cross-correlations; and iii) remove the potential common influences from other factors. Therefore, the new correlation-detection system can be used to objectively determine on which time scale, during which time period, two time series of interest are significantly correlated. This ability is especially important in the research of complex systems, as the effects of nonstationarity and nonlinearity can to some extent be removed. Based on the information provided by the new methods, we can make more detailed diagnosis on the unknown dynamics of complex system, and also design better models, such as the hierarchical model, where factors of different time scales can be better organized.

In this study, we used two climatological examples to illustrate the advantages of the new correlation detection system. We suggest, that those methods offer new opportunities and possibilities for applications in other natural sciences with long data series, ecology, economy, sociology and other research fields where nonlinear interactions of multi-factors exist. By studying how the factors are correlated with each other (on which time scale, during which time period), we may have better chance to interpret the outputs of the complex system, and provide more accurate estimations for future conditions. Therefore, the new methods proposed in this study provide new perspectives in the detection of correlations in many scientific fields.

## Data and Methods

### Data

In this study, we performed two case studies. In the first case, the Reconstructed North Atlantic Oscillation (RNAO) index are downloaded from the National Oceanic & Atmospheric Administration (NOAA, http://www.esrl.noaa.gov/psd/gcos_wgsp/Timeseries/RNAO/), and the Global sea level (GSL) data are downloaded from Permanent Service for Mean Sea Level (PSMSL, http://www.psmsl.org/products/reconstructions/jevrejevaetal2008.php). In the second case, to compare with the results reported in^20^, the Nino3 Sea Surface Temperature Anomaly (Nino3-SSTA) and the Pacific Decadal Oscillation (PDO) index are downloaded from the National Oceanic & Atmospheric Administration (NOAA, http://www.esrl.noaa.gov/psd/data/climateindices/), and the Summer Rainfall over the middle-lower reaches of the Yangtze River (SRYR) are calculated based on the monthly observations of 17 stations. The locations of the 17 stations are available in^20^. The data ranges from 1951 to 2012, but for Nino3-SSTA and PDO, we only consider the previous winter-time values (averaged over December, January, and February).

## Methods

### Introduction of DCCA/DPCCA

In this section, we will first introduce the methods DCCA/DPCCA.

Suppose we have *m* time series 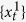, 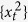, 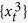, ···, 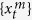, where *t* = 1, 2, 3, ···, *N*, represents the time points in each series. Each time series can be considered as a random walk, and we can define the so called profile as:


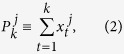


where *j* = 1, 2, 3, ···, *m*, *k* = 1, 2, 3, ···, *N*. To calculate cross-correlations on different time scales, one needs to first divide the entire profile into *N* − s^*^ overlapping boxes. Each box *i* (*i* = 1, 2, 3, ···, *N* − s^*^) contains *s* = s^*^ + 1 values, starts at *i* and ends at *i* + s^*^. Here, *s* = s^*^ + 1 represents the time scale, which is the size of each box. By varying *s*, we can obtain results for different time scales. Before calculating cross-correlations, “local trend” 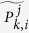 (*i* ≤ *k* ≤ *i* + s^*^) is determined by using a polynomial fit, and the “detrended walk” are calculated as the difference between the original profile and the local trend, as:


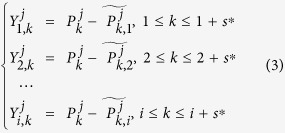


For climatic records, normally the polynomial fit of second order (*n* = 2) is enough. Accordingly, the minimum time scale *s* should be larger than *s* = *n* + 2 = 4. In other words, the varying range of the time scale *s* should be *n* + 2 < *s* < *N*, where *n* is the order of the polynomial fit. To study the correlations on different time scales without getting a temporal evolution information, we can splice the detrended residual series from each boxes into one time series,


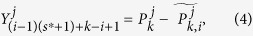


therefore, for each time series 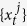, we have one detrended residual series, 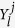, *l* = 1, 2, 3, ···, (*N* − *s*)(*s* + 1). By calculating the covariance between any two residuals,


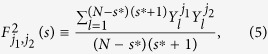


where *j*_1_, *j*_2_ = 1, 2, 3, ···, *m*, we can obtain a covariance matrix,


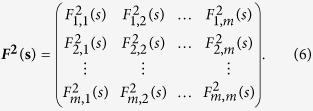


Obviously, according to^17^, the cross-correlation levels between any two time series, 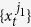 and 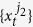, can be estimated as,


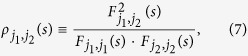


and a coefficients matrix can further be obtained as,


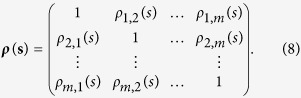


Where 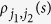 (*j*_1_ ≠ *j*_2_) ranges from −1 to +1, and represents the level of cross-correlation on time scales of *s*. This is the so called DCCA cross-correlation coefficients *ρ*_*DCCA*_.

Since 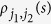 only shows the relations between time series 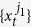 and 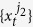. It may provide spurious correlation information if the two time series are both correlated with other signals. Therefore, to exclude the possible influence of other time series, one need to further combine the partial-correlation technique with the calculations above, as following,


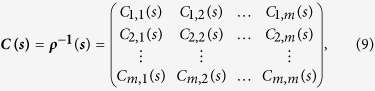


one first calculate the inverse matrix of ***ρ***(***s***). For any two time series 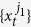 and 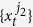, the partial-cross-correlation level can thus be determined as,


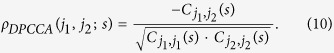


where the coefficients *ρ*_*DPCCA*_(*j*_1_, *j*_2_; *s*) can be used to characterize the “intrinsic” relations between the two time series, with possible influences of other time series removed. By changing *s*, similar to the DCCA cross-correlation coefficient *ρ*_*DCCA*_, we can further estimate the partial cross-correlation levels on different time scales. This, is the so called DPCCA cross-correlation coefficient *ρ*_*DPCCA*_.

### Introduction of TDCCA/TDPCCA

If the temporal evolution of the correlation is of interest, we need to study the detrended residual series 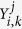 on different time windows (boxes). For a given time scale *s*, the detrended residual series can point to time points as following,


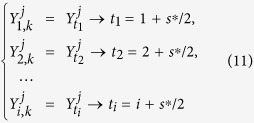


Then for different time series 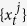, we can get a new matrix, where the horizontal direction represents the temporal evolution, and the vertical direction shows the different time series,


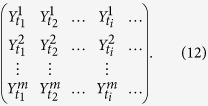


According to equations (5) and (7), one can calculate the cross-correlation coefficients between each pair of time series (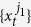 and 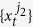) for different time points, as shown below,


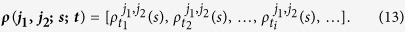


By varying the time scale *s*, one can obtain the cross-correlation information between 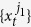 and 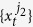 on different time scales and for different time points (Fig. 4). This, is the so called TDCCA, which is generalized from DCCA.

As for TDPCCA, we need to combine the partial-correlation technique with TDCCA by using equations (9) and (10). To do this, we can simply focusing on each time point, for example, *t*_*i*_. From TDCCA, one can easily obtain a correlation matrix on time point *t*_*i*_, which is quite similar to the matrix obtained from DCCA, see equation (8),


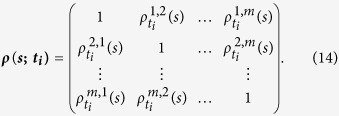


According to equation (9), it will be easy to calculate the inverse matrix of ***ρ***(***s***; ***t***_***i***_) at time point *t*_*i*_, as shown below,


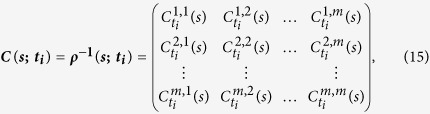


For any two time series 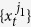 and 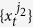, the partial-cross-correlation level at time point *t*_*i*_ then can be determined as,


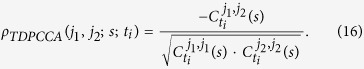


By changing the time point *t*_*i*_ and the time scale *s*, one can then obtain the so called “intrinsic” cross-correlations between two time series on different time scales, and for different time points. As shown in Fig. 6, we name this results “temporal evolution of cross-correlations”, the so called TDPCCA.

DCCA/DPCCA, TDCCA/TDPCCA together can be considered as a new system for correlation detecting. With this new system, one can tell on which time scale, in which time period, the two considered time series are significantly correlated. To apply this system, we suggest to follow the steps given in the “Conclusion and Discussion” section, where one needs to first use TDCCA/TDPCCA to check on which time scale *s*, during which time period, the sign of correlations for the two time series of interest can keep unchanged for a long time (longer than the time scale *s*). Then by applying DCCA/DPCCA to the corresponding time period, with the Monte-Carlo test, we can confirm whether the two time series are significantly correlated on this typical time scale, and over this specific time period. Accordingly, we could finally determine the correlation details.

### ARFIMA model

In this study, we applied the autoregressive fractionaly integrated moving average model (ARFIMA) to model long-term persistent data, as following^43^,


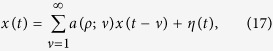


where *η*(*t*) denotes an independent and identically distributed (i.i.d) Gaussian noise, *a*(*ρ*; *ν*) is statistical weights defined by^43^


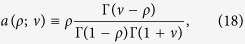


and Γ(*ν*) denotes the Gamma function. In this model, *ρ* is a free parameter ranging from 0 to 0.5, which determines how strong long-term persistence the simulated time series will have. In this study, we modeled the persistence properties of GSL by setting *ρ* = 0.4^44^, and generated artificial data for the calculation of Fig. 7.

## Additional Information

**How to cite this article**: Yuan, N. *et al*. A novel way to detect correlations on multi-time scales, with temporal evolution and for multi-variables. *Sci. Rep.* **6**, 27707; doi: 10.1038/srep27707 (2016).

## Figures and Tables

**Figure 1:**
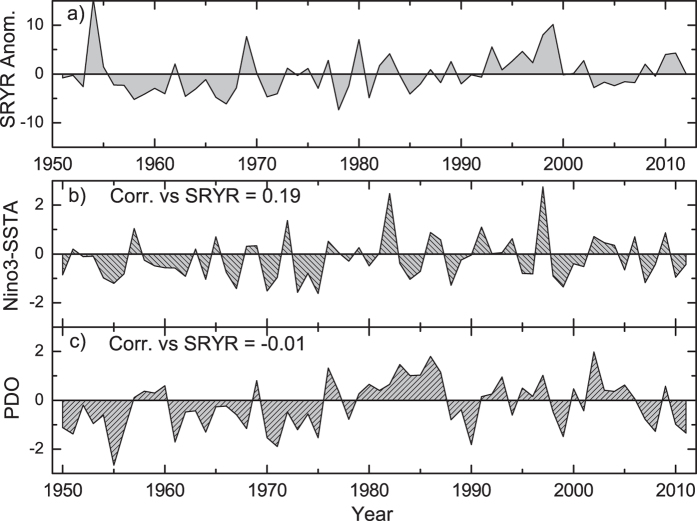
Three time series for illustration. (**a**) Summer Rainfall anomalies over Yangtze River (SRYR) during the period of 1951–2012, (**b**) previous winter-time Sea Surface Temperature anomalies over the Nino3 region (Nino3-SSTA), (**c**) previous winter-time PDO index from 1951–2012.

**Figure 2:**
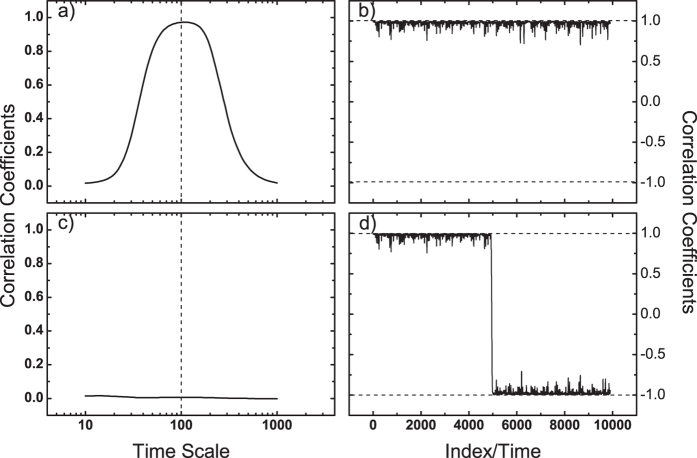
An illustration of the importance in studying temporal evolution of correlation. Three artificial time series are generated according to equation (1). (**a,c**) are the DCCA results for pair {*x*_1_, *x*_2_} and pair {*x*_1_, *x*_3_}, respectively. As expected, for pair {*x*_1_, *x*_2_}, significant correlations are found on time scale of 100, while for pair {*x*_1_, *x*_3_}, no correlations are found over all time scales. (**b,d**) show the temporal evolution of the correlation on time scale of 100, for pair {*x*_1_, *x*_2_} and pair {*x*_1_, *x*_3_}, respectively. One can see the reason why no correlations are found in (**c**) is, that there are opposite correlations over time for the pair {*x*_1_, *x*_3_} on time scale of 100.
